# Second Periprosthetic Joint Infection Caused by *Streptococcus dysgalactiae*: How Genomic Sequencing Can Help Defining the Best Therapeutic Strategy

**DOI:** 10.3389/fmed.2020.00053

**Published:** 2020-02-21

**Authors:** Truong-Thanh Pham, Vladimir Lazarevic, Nadia Gaia, Myriam Girard, Abdessalam Cherkaoui, Domizio Suva, Jacques Schrenzel

**Affiliations:** ^1^Division of Infectious Diseases, Department of Medicine, Geneva University Hospitals, Geneva, Switzerland; ^2^Division of Orthopedics and Trauma Surgery, Department of Surgery, Geneva University Hospitals, Geneva, Switzerland; ^3^Genomic Research Laboratory, Division of Infectious Diseases, Department of Medicine, Faculty of Medicine Geneva, Geneva University Hospitals, Geneva, Switzerland; ^4^Bacteriology Laboratory, Department of Diagnostic, Geneva University Hospitals, Geneva, Switzerland

**Keywords:** periprosthetic joint infection, PJI, *Streptococcus dysgalactiae*, next-generation sequencing, NGS

## Abstract

Primary and revision arthroplasties are increasing worldwide, as are periprosthetic joint infections (PJI). The management of PJI requires surgery, the strategy of which is dictated by the acute or chronic nature of the infection, with an exchange of the implant in the event of a chronic PJI or in the case of recurrence with the same pathogen. We report the case of a 63-year-old man with two episodes of *Streptococcus dysgalactiae* subsp. *equisimilis* PJI within 9 months. Based on clinical suspicion of an haematogenous PJI, the patient was treated by DAIR (debridement, antibiotics, implant retention), while genomic sequencing revealed two different strains, confirming our hypothesis that no additional surgery was needed. Hence, we report a case where genomic analysis was decisive for the decision of the best therapeutic strategy.

## Introduction

There is an increasing trend for primary and revision arthroplasties in the United States (US) (1) and in Europe (2, 3). The incidence of periprosthetic joint infections (PJI) ranges from 0.5 to 2.0% (4). Twenty-five to thirty-eight percent of knee replacements are due to infections and have been associated with a significant morbidity and a 5-fold increase in mortality at 1 year (4, 5). The management of peri-prosthetic joint infections is challenging and therefore requires a multidisciplinary approach, typically including infectious diseases specialists, microbiologists, and orthopedic surgeons to decide which of the following options may be the best in each case: 1 or 2-stage exchange, DAIR (Debridement, Antibiotic, and Implant Retention), ablation of the prosthesis (Girdlestone procedure for example), arthrodesis or amputation.

Herein, we report two episodes of a prosthetic joint infection due to *Streptococcus dysgalactiae* subsp. *equisimilis* within a 9 month-interval. Genomic sequencing of the two strains showed that the 2 strains were genetically unrelated. The patient was treated by DAIR for the second PJI, hypothesizing an hematogenous infection rather than a recurrence or a chronic infection, and therefore additional surgical intervention was avoided.

## Case Report

We report the case of a 63-year-old man, known for a right Charcot foot, lymphedema associated with venous insufficiency of the right lower extremity and bilateral knee arthroplasties (right knee 6 months prior without complications, left knee 4 years prior). Two days prior to admission, the patient presented with redness of the right knee, pain and increased swelling, fever, and shivering. The patient did not report any trauma before this episode.

He consulted at a private clinic where a blood test showed a C-reactive protein (CRP) at 50.9 mg/L [0–10 mg/L]. An arthrocentesis was performed which revealed 156,660 leucocytes/μL (36% neutrophils), 70,000 erythrocytes/μL, and direct examination showed the presence of gram-positive cocci. A treatment with cefuroxime administered intravenously (IV) was initiated. The next day, the patient's condition deteriorated with hypotension not responding to IV fluid administration. Antibiotic treatment was changed to amoxicillin-clavulanate 2.2 g IV and the patient was transferred to our hospital.

On arrival, the patient was in septic shock and blood tests revealed a CRP at 426.5 mg/L [0–10 mg/L], and an acute kidney failure with creatinine at 355 μmol/L [62–106 μmol/L] (eGFR 15 mL/min/1.73 m^2^). He was promptly taken to the operating room (OR) where a large amount of pus was collected around the prosthesis and a DAIR procedure with exchange of moving parts was performed considering an acute PJI. Cultures of the purulent material obtained in the OR became positive for *S. dysgalactiae* subsp. *equisimilis* and antibiotic therapy was initially changed to ceftriaxone 2 g/day IV and subsequently to penicillin G 4 MioU six times daily, after excluding an infective endocarditis. A second and third look were performed, because of steady increase in CRP on post-operative day (POD) 4: the prosthesis was ablated with the placement of a spacer with decision of a 2-stage exchange. However, abscesses of the right foot were formed requiring a new surgical intervention on POD14 and the treatment was empirically changed to amoxicillin-clavulanate 1,000/200 mg four times daily. Operative samples showed *S. epidermidis* and vancomycin 1 g IV twice daily (bid) was introduced. Clinical response was documented, and antibiotic treatment was subsequently narrowed down to fusidic acid 500 mg orally (PO) three times daily with rifampicin 600 mg PO once daily. He received a total of 6 weeks of antibiotics after the prosthesis was removed, and eventual reimplantation on POD90. Cultures obtained during this surgery were all negative.

Six months after reimplantation, the patient presented with fever, shivering and right knee pain. Upon presentation, he was again in septic shock and on clinical examination he had a warm, red, and swollen right knee with a clear cellulitis extending to the leg. Blood tests showed a leukocyte count of 16,400 cells/μL [4,000–11,000 cells/μL], CRP 310 mg/L [0–10 mg/L]. Arthrocentesis revealed opaque fluid, with 120,338 leukocytes/μL (98% neutrophils), 193,306 erythrocytes/μL, and direct examination showed gram-positive cocci. The patient was quickly taken to the OR where a DAIR was performed and treatment with cefazoline 2 g three times daily IV and vancomycin 1 g twice daily IV was initiated post-operatively. Blood cultures obtained on admission and perioperative cultures became positive for *S. dysgalactiae* subsp. *equisimilis*. Although a chronic, insufficiently treated infection was suspected, in view of the cellulitis with lymphedema and chronic vein insufficiency, the origin of this second episode was considered to be the cellulitis with *S. dysgalactiae* bacteremia and secondary seeding. A DAIR was therefore a valid option, particularly after considering the recent implantation of a revision implant.

In order to confirm the source of this infection (relapse or new infection), we performed a genomic analysis of the strains GE-044 and GE-045 (Table 1) recovered from the periprosthetic purulent aspirate of the first and second episode, respectively. Genomic sequences were generated in the same sequencing run on an Illumina iSeq 100 benchtop system with 2 × 151 cycles. Comparison of sequence contigs revealed that the two strains had an average sequence identity (ANI) (7) of only 98.81%, while each strain showed higher, >99% ANI to several *S*. *dysgalactiae* subsp. *equisimilis* isolates available in the NCBI database (https://www.ncbi.nlm.nih.gov/assembly). The seven MLST alleles of strains GE-44 (*gki*_3, *gtr*_3, *murI*_2, *mutS*_8, *recP*_9, *xpt*_6, *atoB*_6) and GE-45 (*gki*_3, *gtr*_2, *murI*_4, *mutS*_2, *recP*_20, *xpt*_1, *atoB*_3) perfectly matched (100% alignment and 100% identity) those of sequence types ST-20 and ST-55, as revealed using the BIGSdb software at the *S*. *dysgalactiae* MLST website (https://pubmlst.org/sdysgalctiae/) (8). *gki*_3 was the only allele common to both isolates. A single ST-55 strain previously reported in the PubMLST database was collected in Europe (Portugal). Eleven ST-20 type strains already present in the PubMLST database originated from four continents (Asia, Australia, Europe, North America). The results of our genomic analysis suggest a new infection rather than relapse.

**Table 1:** *S. dysgalactiae* strains isolated in this study.

**Strain**	**Isolation date** **(month-year)**	**Total length (nt); number of contigs >500 nt^a^**	**Sequencing depth**	**Best ANI hit;** **strain;** **GenBank assembly accession**	**Sequence type**
GE-044	03-2018	2,067,268; 74	52 ×	99.687; *S*. *dysgalactiae* subsp. *equisimilis* UT_4242_AB; GCA_001682815.1	ST-20
GE-045	12-2018	2,023,504; 75	53 ×	99.957; *S*. *dysgalactiae* subsp. *equisimilis* T642; GCA_002094215.1	ST-55

a *Sequencing data were assembled using SPAdes (6)*.

Antibiotic treatment was transitioned to penicillin G 4 MioU every 4 h IV on day 1 after surgery for 2 weeks with good response and hospital discharge by day 15 on clindamycin 900 mg three times daily PO for 4 additional weeks, for a total of 6 weeks of antibiotic treatment post-surgical intervention. Subsequently, the patient was put under suppressive antibiotic therapy with penicillin V 0.5 MioU twice daily considering the high risk of recurrence and a second severe streptococcal infection within 6 months. At 1 year post-surgical procedure, the patient had no further relapse.

## Discussion

The management of peri-prosthetic joint infections remains challenging and requires consideration of several parameters. In the context of chronic infections, it is preferable to exchange the prosthesis (one- or two-stage approach), because of the presence of a mature biofilm, that may be almost impossible to sterilize without changing the material (9). On the other hand, in the setting of acute infection or when the patient's condition does not allow it (e.g., comorbidities, age, etc.), a DAIR may be preferable (10, 11).

The time between arthroplasty and infection should also be considered, historically divided into early (within the first 3 months), delayed (between 3 and 12 months) and late (>12 months) post-operative infections. For instance, early post-operative infections are usually due to more virulent bacteria, such as *Staphylococcus aureus* and gram-negative bacilli. During the delayed post-operative period, infections are mainly due to less virulent bacterial pathogens, including *Cutibacterium acnes* and *Staphylococcus epidermidis*, among others. Finally, during the late post-operative period infections may be due to low virulent pathogens, but also due to direct inoculation and/or hematogenous seeding in the setting of a concomitant transient or sustained bacteremia (12).

In this case report, despite the isolation of the same type of organism in two episodes of PJI within a period of 9 months, the second episode represented a new infection, as suggested by: (i) the clinical presentation compatible with cellulitis of the leg, favored by chronic lymphedema and venous insufficiency which are known risk factors (13), with bacteremia and consequently a hematogenous PJI and (ii) a genomic analysis, which revealed that the two isolates collected at a 9 month interval were genetically distant. Notably, streptococci are the second most frequent cause of PJI of hematogenous origin after *S. aureus* (14). Therefore, because of the hematogenous origin, a management by DAIR with exchange of moving parts instead of complete exchange of prosthesis was considered, thus a new surgery could be avoided. Next-generation sequencing (NGS) provides better resolution for strain differentiation than other molecular methods. Starting from a cultured isolate, or directly from a clinical specimen, genomic sequence of a pathogen may be obtained and analyzed in <30 h (15) when NGS is implemented as a routine procedure. While direct NGS of a clinical specimen greatly reduces the overall turnaround time (by bypassing the need to culture bacteria), the completeness of the genomic sequence obtained for a given pathogen depends not only on the sequencing depth but also on the efficacy of human DNA removal, bacterial load and the nature of infection (monomicrobial vs. polymicrobial or monoclonal vs. polyclonal). In the present research study, the reagent cost of NGS for genomic testing of two samples was ~700 CHF. This figure does not take into account labor costs and infrastructure (NGS and computational) investment. Implementing of NGS in routine diagnostics may substantially reduce the cost per sample by multiplexing more samples in the same sequencing run; labor costs for data analysis and interpretation will depend on the computational resources, the level of automation of bioinformatics processes, the availability of curated databases and the nature of each individual clinical case.

Suppressive antibiotic therapy was administered in this patient presenting with a second *S. dysgalactiae* infection with the necessity of hospitalization in the intensive care unit. According to Thomas et al., penicillin as a suppressive treatment is effective in preventing recurrent cellulitis, although the protective effect gradually decreases once treatment is stopped (16). Furthermore, streptococcal PJIs treated with DAIR have a high recurrence rate, with 42.1% treatment failure (17). This high rate of failure has led some experts to suggest suppressive treatment for at least 1 year for streptococcal infections. Additional data suggest that suppressive antibiotic therapy may be associated with better outcomes in streptococcal PJI (93 vs. 57%, *p* = 0.002, median follow up of 13 months, range 0.5–111 months) (18). Although long-term follow-up data are not available for this patient, he remained without recurrent episodes for at least 1 year on low-dose penicillin secondary suppressive prophylaxis treatment.

## Conclusion

Identification of the same organism in recurrent PJIs, found in 31% of cases (19) is commonly considered as indicative of either chronic or relapsed infections. Our case report, based on NGS data analysis, illustrates the fact that recurrent infections due to same bacterial pathogens could represent a new infection, which could have significant implications in the management of those patients. A multidisciplinary approach, including infectious disease, microbiology, and orthopedic surgery specialists, to take into account the case as a whole and determine the best management strategy is required. Genomic sequencing, by the virtue of precisely determining genetic relatedness of sequentially collected clinical isolates from the same patient, can occasionally be the determinant factor for choosing the best approach. In fact, this case report is the first to demonstrate a clear contribution that genomic sequencing can make to the strategy of peri-prosthetic joint infections management.

## Data Availability Statement

Assembly contig fasta files of stains GE-044 and GE-045 were uploaded to the *S*. *dysgalactiae* PubMLST BIGSdb database (https://pubmlst.org/sdysgalctiae/) (8).

## Ethics Statement

According to hospital protocol, no formal ethics approval was required. The patient agreed and provided written informed consent for publication of this case report.

## Author Contributions

TP, VL, DS, and JS analyzed and interpreted patient data. NG, MG, and AC performed the experiments. NG and VL analyzed the genomics data. TP and JS wrote the manuscript. All authors read and approved the final manuscript.

### Conflict of Interest

The authors declare that the research was conducted in the absence of any commercial or financial relationships that could be construed as a potential conflict of interest.
